# Early Stage and Locally Advanced Nasopharyngeal Carcinoma Treatment from Present to Future: Where Are We and Where Are We Going?

**DOI:** 10.1007/s11864-023-01083-2

**Published:** 2023-05-05

**Authors:** Juan Jose Juarez-Vignon Whaley, Michelle Afkhami, Sagus Sampath, Arya Amini, Diana Bell, Victoria M. Villaflor

**Affiliations:** 1 Health Science Research Center, Faculty of Health Science, Universidad Anahuac Mexico, State of Mexico, Mexico City, Mexico; 2 Department of Pathology, City of Hope Comprehensive Cancer Center, Duarte, CA USA; 3 Department of Radiation Oncology, City of Hope Comprehensive Cancer Center, Duarte, CA USA; 4 Department of Medical Oncology, City of Hope Comprehensive Cancer Center, 1500 East Duarte Road, Duarte, CA 91010 USA

**Keywords:** Nasopharyngeal carcinoma, Locally advanced disease, Management NPC, Concurrent chemotherapy, Induction chemotherapy, Adjuvant chemotherapy

## Abstract

Nasopharyngeal carcinoma (NPC) is a rare malignancy, endemic in China, that is commonly diagnosed in locally advanced scenarios. Its pathogenesis is strongly associated with Epstein-Barr virus (EBV), an infection for which measuring EBV plasma DNA levels has helped as a prognostic factor guiding treatment options, including a stronger treatment in those with high titers. Additionally, tobacco and alcohol are often implicated in EBV-negative patients. The local disease is treated with radiotherapy alone, preferentially intensity modulated radiotherapy. For locally advanced disease, the backbone treatment is concurrent chemoradiotherapy with the ongoing research dilemma being adding adjuvant chemotherapy or induction chemotherapy. The ongoing research is focused not only on identifying patients that will benefit from adjuvant or induction chemotherapy, but also on identifying the best chemotherapeutic regimen, regimen alternatives to diminish toxicity, the role that immune checkpoint inhibitors play, and the use of molecularly guided treatment targeting patients with NPC whether driven by EBV or tobacco and alcohol. Knowing the precise oncogenesis of NPC not only offers a better understanding of the role that EBV plays in this tumor but also helps create targeted therapies that could potentially block important pathways such as the NF-κB pathway. Much is yet to be done, but the prognosis and management of NPC patients have changed drastically, offering precise treatment methods and excellent control of the disease, even in locally advanced scenarios.

## Introduction

Nasopharyngeal carcinoma (NPC) is a rare malignancy with age-standardized rates worldwide below 1 per 100,000 person-years [1]. In 2021, a total of 133,000 new cases were diagnosed, and 80,000 deaths occurred due to nasopharyngeal carcinoma [2]. However, NPC is common in Southern China, especially in the Cantonese population, with an increased incidence rate of up to 50-fold between Northern and Southern China [3]. NPC has a 2–3 times higher incidence in males compared to females, with a higher incidence peak between 50 and 59 years of age [4].

The World Health Organization (WHO) classifies NPC into three subtypes [5, 6]: (1) squamous keratinizing cell carcinoma associated with tobacco exposure; (2) non-keratinizing squamous cell carcinoma potentially viral (EBV and human papillomavirus [HPV]) or tobacco-related; (3) undifferentiated or poorly differentiated carcinoma (lymphoepithelial variants) that are EBV related. NPC’s most common histology in high-incidence areas is non-keratinizing/undifferentiated subtypes, while in low-incidence areas, most cases belong to conventional keratinizing squamous cell carcinoma [1]. NPC is multifactorial, with the most important risk factor being EBV infection, common in infants and young adults in endemic regions. Other risk factors include salt-preserved foods, tobacco and alcohol use, and lately anecdotal associations with HPV infection, especially in non-endemic regions [7, 8].

NPC does not have a specific clinical presentation; however, headache, facial numbness, neck mass, nasal obstruction, epistaxis, and otitis media, together with risk factors, should raise suspicion for NPC. Unfortunately, due to its location and vague clinical presentation, most patients remain asymptomatic and are diagnosed with locally advanced disease. NPC is diagnosed preferentially with an endoscope-guided biopsy of the primary tumor, followed by adequate staging and pretreatment plasma EBV-DNA levels [9, 10].

As most patients are diagnosed in locally advanced scenarios, the development of new therapeutics is essential. In this manuscript, we focus on the current landscape of treatment and multiple clinical trials where the latest advances have been made. Understanding state-of-the-art treatments for NPC in local and locally advanced diseases, how it has changed, and future investigations are crucial.

## Treatment for local disease

Local disease, classified as early stage I disease, encompasses patients with a tumor limited to the nasopharynx, or adjacent oropharynx, nasal cavity, without parapharyngeal involvement (T1), and no lymph node (N0) or distant metastasis (M0). Treatment at this stage has had limited advances regarding management, as international consensus establishes treatment with radiation therapy (RT) alone, as they are radiosensitive, have a limited surgical approach, and RT achieves excellent local control [11]. Precisely, intensity modulated RT (IMRT) is the mainstay treatment at this stage.

### IMRT

Radiotherapy techniques have evolved, with IMRT demonstrating better results than 2D RT (two-dimensional radiotherapy) and 3D RT (three-dimensional radiotherapy). IMRT delivers high doses of radiation to the tumor, reduces toxicity to surrounding healthy tissue, and enables dose escalation [12]. A retrospective study demonstrated a 5-year distant failure-free rate of 85% for IMRT compared to 81% for 3D RT and 78% for 2D RT, as well as a decreased toxicity rate (1.8% vs 3.5% vs 7.4%) [13]. IMRT also improves the quality of life and spares parotid involvement in the early stages compared to conventional radiotherapy [14]. A phase II trial by Lee et al. evaluated the efficacy of IMRT in locoregionally advanced disease, including nine patients with stage I disease treated with IMRT only [15]. For these nine patients, none of them had a locoregional failure at a median follow-up of 2.6 years. This established IMRT alone as the standard of care for stage I NPC, providing the longest local recurrence-free survival.

### Is there a role for surgery?

Surgical approaches are not the mainstay initial treatment for early disease, due to complex anatomical locations, access, and neurovascular damage. Surgery may be indicated in residual node disease post-radiation therapy and isolated neck recurrence. Its use as a first-line treatment over IMRT is not recommended. Liu et al. compared the use of IMRT vs minimally invasive surgery via an endoscopic nasopharyngoscopy in 339 patients with stage I NPC, of whom only 10 underwent surgical approach [16]. The study demonstrated similar survival outcomes with a 100% 5-year overall survival (OS), local relapse-free survival, regional relapse-free survival, and distant metastasis-free survival (DMFS), compared to 99.1%, 97.7%, 99.0%, and 97.4%, respectively for those treated with IMRT. The major emphasis of the study was regarding the impact on quality of life and lower medical costs for those treated with the minimally invasive surgical approach. This study offers the minimally invasive surgical approach as an alternative strategy, especially for patients who refuse IMRT due to toxicity concerns. However, performing a surgical approach over IMRT must be discussed with a multidisciplinary team.

## Treatment for locally advanced disease

Locally advanced scenarios (stage III to IVA) together with metastatic scenarios (IVB) are the most common stages at which patients with NPC are diagnosed. Patients with the locally advanced disease include those cases involving at least one lymph node or bilateral retropharyngeal nodes (N1) and/or tumor extension into the parapharyngeal space (T2), bony structures (T3), or intracranial extensions (T4) but have not metastasized distantly (M0). Stage II (T1/T2, N1 or T2 N0), also known as intermediate disease, is not considered an advanced scenario, and treatment is based on RT with or without concurrent chemotherapy depending on the risk of disease recurrence.

### Role of RT

IMRT must be the preferred mode, as mentioned previously. However, for locally advanced diseases, RT alone is not an option, as treatment must include concurrent chemoradiotherapy (CCRT). Important changes that have been done in RT for NPC compared to the past include potentially avoiding bilateral whole-neck RT where selective neck radiation in the node-negative neck can be offered, which is limited to retropharyngeal lymph nodes and levels II, III, and VA [17]. The reasoning for sparing nodes in the lower neck and IB level comes from the common pathway of lymphatic spread, with retropharyngeal and level II neck nodes being the most commonly affected. Avoiding RT at these nodes, in particular IB, is useful as it reduces xerostomia. IMRT has been demonstrated to achieve a 5-year local control of 90% for T3 disease and 74–80% for T4 [18, 19].

Future advances in the area of radiotherapy include accurate methods to delineate gross tumor volume, appropriate identification of margins, and clinical target volume, making these characteristics the objective of precision RT which is being accomplished by computer tomography, nowadays known as image-guided RT. These advances have the goal of limiting tissue damage and accurately identifying the radiated area [11].

### Concurrent chemoradiation (CRT)

Concurrent CRT is recognized as the backbone for treating locally advanced NPC as established by the phase III United States Intergroup 0099 trial led by Al-Sarraf et al., where patients received chemoradiation with cisplatin followed by adjuvant chemotherapy (AC) with cisplatin plus fluorouracil or RT alone [20]. The median progression-free survival (PFS) at 15 months was not reached for the chemoradiotherapy group compared to the RT alone group, and the 3-year PFS rate was 69% vs 24% respectively. Similar results were observed for OS, in which the median OS was not reached for the CRT group and the 3-year survival rate was 78% vs 47%, respectively. Multiple clinical trials have demonstrated the benefit of concurrent CRT vs RT alone for both 5-year OS and 5-year PFS [15, 21–23]. Taking these studies into consideration, the 5-year OS and 5-year PFS were 75.3% and 65.9%, respectively for concurrent CRT vs 64.1% and 53.7% for those with RT alone. A meta-analysis of 4798 patients in which most of whom were stage III-IVB, an absolute OS benefit of 6% was observed at 5 years and 8% at 10 years [24]. Another meta-analysis focused on endemics demonstrated a benefit for concurrent CRT at 2, 3, and 5 years for OS, confirming the superiority of concurrent CRT over RT [25]. Similarly, a meta-analysis performed in Chinese patients proved that concurrent CRT improved the overall response rate, complete response rate, and had longer OS compared to RT; however, concurrent CRT had a higher degree of hematologic toxicities and mucositis [26]. On the other hand, a meta-analysis in non-endemic populations found that concurrent CRT improved both OS (10-year OS 59% vs 51%) and PFS (10-year PFS 52% vs 44%) [27].

While it is well established that the backbone treatment for locally advanced NPC is CRT, questions regarding the addition of induction chemotherapy (IC) or adjuvant chemotherapy (AC) are the main objective of the research, coupled with the role of targeted therapies, predictive biomarkers, and reducing toxicity [28, 29].

### Adjuvant (AC) vs induction chemotherapy (IC). Which one is better?

The question regarding AC vs IC is the most debatable and studied aspect of the treatment of locoregionally advanced NPC. AC has been recognized as the standard of care since 1998 when cisplatin plus fluorouracil was the recommended regimen [20]. Additional trials validated these findings [30, 31]. One clinical trial confirmed these results, demonstrating a benefit for 2- and 3-year OS rates in favor of AC [30]. Similarly, control trials in endemic and non-endemic regions proved the survival benefit of AC with no increase in late toxicities [31–33]. A meta-analysis demonstrated that concurrent CRT followed by AC improved 10-year OS by 14% [27]. Prognostic and predictive factors such as EBV-DNA load are studied. Twu et al. demonstrated that AC reduced distant failure and improved OS in patients with persistently detectable EBV-DNA levels after concurrent CRT. Patients with persistent detectable EBV-DNA levels experience a lower tumor relapse with AC (45.5%) vs those without (71.2%) [34].

Studies have also demonstrated the opposite results. A clinical trial comparing RT alone vs AC (vincristine, cyclophosphamide, and doxorubicin) demonstrated no significant differences at 48 months in terms of relapse-free survival and OS [35]. Later on, a phase III study demonstrated no significant benefit for OS (60.5% RT vs 54.5% AC) and relapse-free survival (49.5% RT vs 54.4% AC) [36]. A recent phase III multicenter trial in the Chinese population comparing concurrent CRT + AC (cisplatin and fluorouracil) vs concurrent CRT alone demonstrated at 37.8 months and 68.4 months no significant survival benefit or difference regarding toxicities [37]. Similarly, a meta-analysis demonstrated no significant differences between concurrent CRT plus AC and concurrent CRT alone for all endpoints [38].

IC may be recommended for patients with locally advanced disease. The first study to report a benefit in OS reported a 26.5% improvement in 3-year OS by adding cisplatin + docetaxel [39]. Research comparing IC followed by concurrent CRT vs concurrent CRT alone is vast, with trials demonstrating a benefit for IC, including a phase III trial in locoregionally advanced EBV + NPC comparing gemcitabine + cisplatin + CRT vs concurrent CRT alone [40]. At a median follow-up of 42.7 months, a benefit in both 3-year recurrence-free survival (85.3% vs 76.5%) and 3-year OS (94.6% vs 90.3%) in favor of IC was reported [40]. Additionally, the IC reported a 9.2% incidence of grade 34 toxicity compared to 11.4% for concurrent CRT. The follow-up study at 70 months reported an improved 5-year OS (88% vs 79%), failure-free survival (FFS) (81% vs 67%), DMFS (90% vs 78%), and locoregional free survival (88% vs 83%) in favor of IC [41]. This study highlights the importance of Epstein-Barr virus (EBV) DNA load for guiding individualized treatment, in which patients with DNA loads < 4000 copies/mL do not benefit from IC compared to those with a higher viral load. Mané et al.’s meta-analyses identified that the addition of IC to concurrent CRT provides a significant benefit in OS and PFS compared to concurrent CRT alone [42].

Few studies have directly compared IC vs AC, with conflicting evidence being reported. One study demonstrated IC improves OS, PFS, DMFS, and locoregional recurrence-free survival [43]. On the other hand, another meta-analysis demonstrated that AC achieves a higher survival benefit, but IC had greater distant control [44]. A propensity score-matched analysis demonstrated IC + concurrent CRT improved survival in T3 and N2 disease, with AC improving local control but not survival outcomes in T4 disease [45].

It is clear that more work is needed to determine if there is a benefit in favor of IC based on the conflicting evidence. On one hand, IC could offer better tolerability, easier delivery, and overcome poor tolerance, but IC could affect the definitive CRT dose. Based on current evidence, concurrent CRT with cisplatin with either adjuvant or induction chemotherapy is the treatment of choice. The NRG HN001 trial evaluates the non-inferiority of the omission of AC in low-risk, locally advanced NPC patients.

EBV-DNA levels have been demonstrated to be a determining factor in both IC (favoring those with high viral loads) and identifying patients in need of AC (favoring those with persistently elevated levels). A clinical trial directly comparing them could be difficult to perform; therefore, a direct comparison should focus on answering a specific outcome and future trials in studying the role of EBV-DNA levels in those in need of AC.

The following table (Table 1) summarizes the most important clinical trials highlighting the benefit of concurrent CRT, AC, and IC and their impact on patient survival.

**Table 1:** Therapeutic Benefit Summary of Concurrent Chemotherapy, Adjuvant Chemotherapy, and Induction Chemotherapy in Locoregionally Advanced Nasopharyngeal Carcinoma

Trial	WHO histology	Experiment vs control	OS	PFS	MFS	FFS	DFS
Concurrent chemoradiotherapy (CCRT)							
X. Wu [21]	II and III	RT vs CCRT	60.2% vs 73.2% (5-year)	N/A	63.0% vs 74.7% (5-year)	N/A	N/A
A.W.M. Lee [22]	II and III	RT vs CCRT	64% vs 68% (5-year)	53% vs 62% (5-year)	N/A	55% vs 67% (5-year)	N/A
J. Ching Lin [46]	I, II, and III	RT vs CCRT	54.2% vs 72.3% (5-year)	53.0% vs 71.6% (5-year)	69.9% vs 78.7% (5-year)	N/A	72.6% vs 89.3% (5-year)
A.T.C. Chan [47]	I, II, and III	RT vs CCRT	N/A	69% vs 76% (2-year)	N/A	N/A	N/A
A.W.M. Lee [48]	II and III	RT vs CCRT	78% vs 78% (3-year)	61% vs 70% (3-year)	N/A	62% vs 72% (3-year)	N/A
L.Zhang [23]	II, II, and III	RT vs CCRT	77% vs 100% (2-year)	N/A	80% vs 92% (2-year)	N/A	N/A
X. Wu and A.T.C Chan [21, 49]	II and III	RT vs CCRT	60.2% vs 73.2% (5-year)	52.1% vs 60.2% (5-year)	63.0% vs 74.7% (5-year)	N/A	N/A
Concurrent + Adjuvant							
M. Al-Sarraf [20]	I, II, and III	RT vs CCRT + A	47% vs 78% (3-year)	24% vs 69% (3-year)	N/A	N/A	N/A
J. Wee [30]	II and III	RT vs CCRT + A	65% vs 80% (3-year)	N/A	N/A	N/A	53% vs 72% (3-year)
Y. Chen [32]	II and III	RT vs CCRT + A	62% vs 72% (5-year)	57% vs 68% (5-year)	N/A	62% vs 72% (5-year)	N/A
A.W.M. Lee [33]	II and III	RT vs CCRT + A	49% vs 62% (10-year)	42% vs 56% (10-year)	N/A	50% vs 62% (10-year)	65% vs 68% (10-year)
L. Chen [37]	II and III	CCRT vs CCRT + A	80% vs 83% (5-year)	N/A	N/A	71% vs 75% (5-year)	80% vs 85% (5-year)
Y. Chen [32]	II and III	RT vs CCRT + A	62% vs 72% (5-year)	57% vs 68% (5-year)	N/A	62% vs 72% (5-year)	71% vs 80% (5-year)
Radiotherapy + adjuvant							
C.W. Twu [34]	II and III	RT vs RT + A	28.7% vs 71.6% (5-year)	28.7% vs 62.9% (5-year)	34.6% vs 71.9% (5-year)	84.2% vs 93.1% (5-year)	N/A
A. Rossi [35]	II and III	RT vs RT + A	67.3% vs 58.5% (2-year)	55.8% vs 57.7% (2-year) (RFS)	N/A	N/A	N/A
K.H. Chi [36]	II and III	RT vs RT + A	60.5% vs 54.4% (5-year)	49.5% vs 54.4% (5-year) (RFS)	N/A	N/A	N/A
Induction + CCRT							
E.P. Hui [39]	I, II, and III	CCRT vs I + CCRT	67.7% vs 94.1% (3-year)	59.5% vs 88.2% (3-year)	N/A	N/A	N/A
Y. Zhang [40]	II and III	CCRT vs I + CCRT	90.3% vs 94.3% (3-year)	76.5% vs 85.3% (3-year)	N/A	N/A	84.4% vs 91.1% (3-year)
Y. Zhang [41]	II and III	CCRT vs I + CCRT	78.8% vs 87.9% (5-year)	N/A	N/A	67% vs 81% (5-year)	78% vs 90% (5-year)
G. Fountzilas [50]	I, II, and III	CCRT vs I + CCRT	71.8% vs 66.6% (3-year)	63.5% VS 64.5% (3-year)	N/A	N/A	N/A
Induction + RT							
D.T. Chua [51]	II and III	RT vs I + RT	71% vs 78% (5-year)	42% vs 48% (3-year) (RFS)	N/A	N/A	N/A

*OS*, overall survival; *PFS*, progression-free survival; *RFS*, recurrence-free survival; *MFS*, metastasis-free survival; *FFS*, failure-free survival; *DFS*, disease-free survival; *RT*, radiotherapy; *CCRT*, concurrent chemoradiotherapy; *I* + , induction chemotherapy; + *A*, adjuvant chemotherapy; *N/A*, not available

## What is the best chemotherapy regimen?

### Concurrent CRT

Cisplatin is still the recommended agent of choice for concurrent CRT [20, 52, 53]. Studies focus on analyzing the efficacy and toxicity regarding the administration method as weekly or bolus dosing. A phase II trial compared weekly vs triweekly cisplatin therapy concurrently with RT and demonstrated that weekly cisplatin improved quality of life with no differences regarding 3-year PFS (64.9% vs 63.8%) or grade 3–4 toxicities (47.2% vs 39.3%) [54]. A separate phase III trial compared bolus cisplatin therapy (once every 3 weeks) vs weekly cisplatin concurrently with IMRT. There was a benefit regarding lower toxicities (55.8% vs 66.3%), especially hematologic and auditory, in favor of bolus cisplatin, with no differences in 3-year FFS (85% vs 86%) [52]. It is important to have alternatives for platinum-ineligible patients (e.g., renal failure), but unfortunately, few studies exist. The two alternative options that have been studied include carboplatin and oxaliplatin. Carboplatin may be an alternative due to its lower toxicity and similar efficacy, as demonstrated by a phase III trial in which carboplatin demonstrated a benefit regarding completion of concurrent CRT (73% vs 53%), a higher percentage of completed AC (70% vs 42%), similar 3-year PFS (60.9% carboplatin vs 63.9% cisplatin), and 3-year OS (79.2% carboplatin vs 77.7% cisplatin) [55]. Another trial compared RT alone vs concurrent CRT with oxaliplatin and demonstrated a benefit on 5-year OS (60.2% vs 73.2%), metastasis-free survival (63.0% vs 74.7%), and similar toxicities in favor of oxaliplatin [21]. Nevertheless, no study has compared oxaliplatin vs cisplatin directly.

### Adjuvant chemotherapy

Cisplatin plus fluorouracil has been the regimen of choice for AC [20, 31, 33]. However, other agents have demonstrated similar efficacy and lower toxicity and are considered part of clinical practice such as carboplatin plus fluorouracil [55]. The use of capecitabine as an AC to concurrent CRT has been studied in metronomic doses. This phase III trial demonstrated an improvement in 3-year FFS (85% vs 76%) and 3-year OS (93% vs 89%) in patients with high-risk locoregionally advanced NPC in favor of adjuvant metronomic capecitabine [56]. This study was performed in the Chinese population, and most patients received IC (docetaxel plus cisplatin). Adjuvant capecitabine has also been studied as a standard dose in stage III-IVB patients with at least one high-risk feature (T3-4 plus N2, any T stage plus N3, pretreatment EBV-DNA ≥ 20,000 copies/mL, tumor volume > 30 cm^2^, PET-CT uptake > 10, or multiple neck node metastases) [57]. At 44.8 months, adjuvant capecitabine demonstrated a benefit in 3-year FFS (87.7% vs 73.3%) and superior disease control over concurrent CRT alone. These two trials demonstrated higher grade 3–4 toxicities compared to concurrent CRT alone, but high compliance rates.

### Induction chemotherapy

The standard regimen for IC consists of gemcitabine plus cisplatin [40, 41]. Nevertheless, different chemotherapeutic regimens have been studied. A phase III trial compared the use of IC based on cisplatin + fluorouracil + docetaxel (TPF) plus concurrent CRT vs concurrent CRT alone [58]. The addition of TPF to concurrent CRT improved 3-year FFS by 8% more than concurrent CRT alone with acceptable toxicity and improved 3-year OS (92% vs 86%). Yang et al. reported their 5-year follow-up results comparing IC with fluorouracil plus cisplatin followed by concurrent CRT vs concurrent CRT alone [59]. At 83 months, a benefit was observed in 5-year disease-free survival (DFS) (73.4% vs 63.1%), DMFS (82.8% vs 73.1%), and 5-year OS (80.8% vs 76.8%) in favor of IC, with no difference in grade ≥ 3 toxicities. Similarly, a phase III multicenter study comparing IC with cisplatin and fluorouracil vs concurrent CRT alone demonstrated a benefit regarding DFS but higher grade 3–4 toxicity [60]. These two studies provide regimens such as TPF and fluorouracil plus cisplatin as alternative options. A phase III GORTEC trial including patients’ stages III–IVB proved the addition of TPF as an induction regimen plus concurrent CRT improved 3-year PFS [61]. Few studies have directly compared different regimen efficacy. Published this year at JAMA Oncology, a phase III trial compared the use of cisplatin + paclitaxel + capecitabine (TPC) vs cisplatin + fluorouracil as IC followed by concurrent CRT [62]. TPC improved FFS (84% vs 69%), DMFS (91% vs 80%), and locoregional relapse-free survival (94% vs 87%) was well tolerable compared to cisplatin plus fluorouracil. Similarly, a recently published trial compared IC with lobaplatin and fluorouracil vs cisplatin and fluorouracil followed by concurrent CRT demonstrating that lobaplatin with fluorouracil resulted in non-inferior survival with fewer toxicity [63]. Even though gemcitabine + cisplatin is considered the preferred regimen, TPC and lobaplatin are acceptable alternatives for this population.

## The role of EBV in therapeutics

The prognosis for local disease patients is promising, with a 5-year OS of 93.2% and an 8-year OS of 85.5% [64]. Retrospective studies have established a 5-year disease-specific survival rate of 94–97% with IMRT alone [65]. The role of pretreatment EBV-DNA levels as prognostic factors impacting the 5-year survival rate in the early stage is well established, with a cut-off point of < 4000 copies/mL having a 91% survival rate and ≥ 4000 copies/mL having a 64% survival rate [66]. These also apply to stage II disease. The role of post-treatment EBV-DNA levels is not well established; however, it may have a role in predicting distant metastasis and stratifying patients for adjuvant therapy or follow-up [67]. Unfortunately, these studies are focused on locally advanced disease (II-IVB), with limited studies specifying the role of post-radiation EBV-DNA levels in patients treated with IMRT alone. One study evaluated the use of post-treatment levels in 385 patients treated with IMRT, of whom 23 patients were stage I [68]. They showed that detectable plasma EBV-DNA post-treatment could be found in patients with undetectable pretreatment levels and was significantly associated with tumor recurrence, especially distant metastasis, regardless of EBV-DNA level status pretreatment.

Plasma EBV-DNA is the most significant prognostic biomarker used to identify patients at risk of failure post-concurrent CRT which are those who should receive AC [69]. Twu et al. demonstrated that adjuvant tegafur-uracil improved distant control and OS in patients with persistently detectable EBV-DNA post-RT vs those without AC [34]. One trial measuring plasma EBV-DNA levels at 6–8 weeks post-RT demonstrated that post-RT plasma levels correlate with locoregional failure, distant metastasis, and death; however, the use of AC did not improve relapse-free survival [70]. Even though a benefit was not observed, failure could have been influenced by the 6-week cut-off point. The previous hypothesis is based on patients having varying periods of time with detectable EBV-DNA plasma levels; one study demonstrated a detectable decreasing rate of 11.5% between 8 and 16 weeks post-RT [71]. The optimal timing to evaluate post-treatment plasma EBV-DNA remains to be identified. Post-RT plasma EBV-DNA can work as an early surrogate maker for long-term survival; patients with detectable levels who experienced subsequent clearance had a greater survival compared to patients with initially undetectable post-RT plasma levels [72]. New technologies for measuring circulating cell-free EBV-DNA (cfEBV DNA) via liquid biopsy have had promising results. A study measuring cfEBV DNA at 3 months post-RT and every 3 to 12 months demonstrated that patients with detectable cfEBV DNA had a higher recurrence rate compared to those with undetectable levels (63.8% vs 8.6%), with high sensitivity, specificity, and accuracy for local, regional recurrence, and distant metastasis [73]. Interestingly, patients with disease recurrence had detectable cfEBV DNA levels 2.3 months prior to clinical and/or radiological recurrence. The ongoing NRG HN001 trial is evaluating individualized treatment based on EBV-DNA. (NCT02135042).

## New systemic and targeted therapies

One of the main areas of research is reducing toxicities via new therapeutic options and identifying prognostic factors such as plasma EBV-DNA.

Targeted therapies including growth factor receptor inhibitors, vascular endothelial growth factor inhibitors, and immunotherapies have completely changed the management of patients with lung, head and neck, and renal cancer. Therefore, studies have evaluated these agents in NPC with most studies focusing on China. A phase II study evaluated the use of concurrent cetuximab-cisplatin and IMRT in locoregionally advanced non-keratinizing NPC demonstrating that its weekly use is a feasible strategy with manageable toxicities and an 86.5% 2-year PFS [74]. Niu et al. evaluated the use of weekly cetuximab plus IMRT with or without chemotherapy and demonstrated it to be effective with a 3-year local failure-free survival (LFFS), region failure-free survival (RFFS), DMFS, PFS, and OS of 86.3%, 83.4%, 83.6%, 70.5%, and 90.9%, respectively [75]. Weekly cetuximab with AC followed by IMRT resulted in 100% local and regional control and 95.2% distant control [76]. Bevacizumab, an antiangiogenic agent, in combination with standard chemoradiation, demonstrated to be a feasible alternative for delaying the progression of subclinical distant disease, achieving 83.7% 2-year locoregional control, 90% DMFS, 74.7% PFS, and 90.9% OS [77]. Results of nimotuzumab (anti-EGF monoclonal antibody) plus chemoradiotherapy vs chemoradiotherapy alone were presented at ASCO 2022, reporting a 5-year OS increase in favor of nimotuzumab (76.9% vs 64.3%) and a good safety profile [78]. Unfortunately, PD1/PDL1 inhibitors are neither standard predictive biomarkers nor immunotherapeutic agents for the locoregional disease, and their use cannot be extrapolated as for other cancers. However, there are two ongoing clinical trials in locoregional NPC analyzing a locally manufactured PD1 monoclonal antibody in China (camrelizumab) and nivolumab (NCT03427827 and NCT03267498).

The multiple ongoing advanced clinical trials will provide more information in the future regarding the best treatment for patients (Table 2).

**Table 2:** Ongoing clinical trials for locoregionally advanced NPC

NCT ID	Phase	Viral status	Purpose/primary objective	Primary outcome
NCT02135042	II/III	EBV positive	Determine whether substituting adjuvant concurrent high-dose cisplatin and 5-FU with gemcitabine and paclitaxel results in superior progression-free survival in patients with detectable plasma EBV DNA Determine whether omitting adjuvant cisplatin and 5-FU will result in non-inferior overall survival as compared with those patients receiving adjuvant cisplatin and 5-FU chemotherapy in patients with undetectable plasma EBV DNA	Overall survival
NCT02874651	II	EBV positive	Non-metastatic NPC patients with residual EBV DNA after curative radiotherapy or chemoradiotherapy. Evaluate the survival in these patients treated with apatinib, an inhibitor of vascular endothelial growth factor receptor, and compare the survival in these patients treated with apatinib vs placebo	Disease-free survival
NCT04453826	III	EBV positive	Demonstrate whether concurrent and adjuvant PD-1 inhibitor (camrelizumab) plus CRT decrease the rate of disease progression and improve survival outcome, in high-risk NPC patients compared to CRT alone	Progression-free survival
NCT03267498	II	N/A	Establish the feasibility of treatment completion of combined CRT-nivolumab regimen followed by adjuvant nivolumab in stage II-IVB NPC patients	Feasibility of treatment completion: rate of completion of adjuvant immunotherapy compared to standard adjuvant cisplatin
NCT04456322	III	EBV DNA < 1500 copy/mL (pretreatment) and undetectable post-I-CT	Determine whether definitive RT plus EGFR blocker (nimotuzumab) is not inferior compared to CCRT in NPC patients with favorable response after I-CT	Progression-free survival
NCT03341936	II	Any HPV status; EBV not mentioned	Evaluate the effectiveness of neoadjuvant nivolumab and lirilumab followed by surgery, followed by adjuvant nivolumab and lirilumab in patients with relapse resectable head and neck tumors	Disease-free survival

*EBV*, Epstein Barr Virus; *HPV*, human papilloma virus; *5-FU*, 5-fluorouracil; *CRT*, chemoradiotherapy; *CCRT*, concurrent chemoradiotherapy; *I-CT*, induction chemotherapy

## Role and impact of viral positivity and targeted therapy

### Epstein-Barr virus (EBV)

Worldwide EBV infection is common, with primary infection occurring in childhood. With NPC being a rare cancer and EBV being a common infection, additional cofactors such as the age of infection and genetic variations most likely mediate the risk of EBV in NPC. A younger age of EBV infection has been suggested as a contributing factor. A Swedish study demonstrated via indicators of early-life EBV infection an association between earlier infection and the development of NPC and an inverse association with infectious mononucleosis [79]. A study in the Chinese population identified four high-risk EBV variant mutations (*BALF2*, *BNFR1*, *V12221*, and *RPMS1*) and their interaction with the immune system as contributing to an increased risk of NPC [80, 81]. These high-risk EBV variants affect the host immune response, promoting an IgA-dependent response. With these antibodies being elevated in NPC patients, they work as the basis for screening high-risk populations and are useful prognostic indicators [82].

The vast analysis of EBV has offered the opportunity to develop new therapeutic advances focused on targeting *EBNA1* and *LMP1*. *EBNA1* plays an important role in viral DNA maintenance, survival, and oncogenic transformation of host cells [83]. Preclinical studies have studied mechanisms to disrupt *EBNA1* function and avoid EBV latency including blockage of DNA binding/dimerization, disruption of the dimer-dimer interface, and targeting the trimer interface [84]. Latent membrane lipoproteins (LMPs), especially *LMP1*, are viral oncoproteins that promote cell proliferation, survival, angiogenesis, and immunosuppressive effects against cancer cells [85]. Inhibition of *LMP1* in NPC cells demonstrated promising results in inhibiting cell proliferation, inducing apoptosis, and suppressing tumor growth [86]. A trial evaluated the efficacy of targeting *LMP1* via a DNAzyme intratumorally in conjunction with radiotherapy [87]. The study demonstrated a higher mean tumor regression rate at 12 weeks in those treated with DNAzyme, an impact on tumor microvascular permeability, and a higher number of samples with undetectable EBV-DNA levels. Other strategies targeting *LMP1* include infusion with autologous EBV-specific cytotoxic lymphocytes, *LMP1*-specific antibodies, and peptide-based inhibitors [88]. Finally, switching the EBV latency state to a lytic cycle promotes cell death and an immune response against EBV-positive cancer cells. This switch can be performed by multiple agents including chemotherapeutic agents, histone deacetylase inhibitors, protein kinase C activators, proteasome inhibitors, and antibacterial agents [84].

EBV analysis may offer a personalized treatment by prognosticating which patients benefit from AC.

### Human papilloma virus (HPV)

EBV is the best-known causative agent in NPC; however, reports have found an association with HPV in certain subgroups. This hypothesis is based on the important tumorigenesis role that HPV plays in head and neck cancers.

NPC WHO types II and III have a strong association with EBV + , but it is less established for WHO type I. A cancer center demonstrated an association with oncogenic HPV in WHO type I NPC [89]. On the other hand, a study by the American Cancer Society associated with NPC WHO type III in Southern China demonstrated 91.9% EBV + , 7.7% HPV + , and 0.6% coinfection. The interesting results were that EBV − /HPV + patients had a lower local recurrence rate compared to EBV + /HPV − (6.4% vs 13.8%), as well as a longer 5-year disease-free survival (89.8% vs 70.8%) and 5-year OS (86% vs 72%) [90]. Different results have been found in non-endemic populations, where HPV + has been correlated with WHO grade II tumors in Caucasian patients and worse outcomes compared to EBV + tumors [91]. These two studies raise awareness that high-risk HPV may play a role in the development of NPC and that prognosis may differ between populations. Unfortunately, the evidence is limited and has conflicting results. For example, one study in the UK found eleven cases with concurrent overexpression of HPV p16, with most cases occurring in white patients with non-keratinizing carcinomas, and none of the HPV + cases showing coinfection with EBV and no OS difference [92]. Similarly, a study in Japan demonstrated a limited number of HPV + NPC type I cases with no impact on survival and concluded no significant role in carcinogenesis [93].

The evidence regarding the role of HPV in NPC carcinogenesis and its clinical significance is limited. So far, it can be assumed that HPV’s role may differ between populations; it is not well established in which WHO histologic type is more prominent, and its presence in the absence of EBV + strongly suggests HPV as a causal factor in NPC development.

## Molecular biology in nasopharyngeal carcinoma

### Germline mutations

Most cases of NPC occur sporadically; therefore, information and identification of germline mutations are limited. Certain germline variants identified in familial NPC cases promote tumor progression and increase the risk of additional somatic mutations. Even though NPC exhibits significant familial aggregation, the susceptible genes are not well identified. *CYLD* somatic mutations play an important role, but germline mutations are associated with familial cylindromatosis characterized by multiple benign head and neck tumors but not NPC [94].

Most of the germline mutations play a role in DNA damage repair mechanisms including *BRCA1/2*, *PRKDC*, *MLH1*, and *KMT2C*, host defense mechanisms genes (*MST1R*), virus infectivity, *BCL2L12*, *NEDD4L*, and signaling pathways (*NOTCH* and *DLL3*) [95–97]. A study analyzed via whole-exome sequencing 13 NPC germline mutations. Missense mutations in the *POLN* gene, *P577L*, *R303Q*, and *F545C* were associated with familial NPC risk, especially EBV + [98]. This occurs because the *POLN* gene is involved in the lytic replication of EBV in NPC cells, promoting viral DNA replication and proliferation. Whole genome sequencing has identified various germline variants that could predispose to NPC which include *MST1R*, *NIPAL1*, *ITGB6*, notch signaling, and pathogenic variants in *JAK2*, *PRDM16*, *LRP1B*, *NIN*, *NKX2-1*, and *FANCE* [99, 100]. The latter genes were associated to intervene in endocytosis and immune-modulating pathways, indicating an important role for immunotherapeutic target drugs. Similarly, multiple germline mutations were identified in EBV + NPC patients, mainly missense and silent mutations in chromosomes 1, 12, and 17 (*GON4L*, *KRAS*, *MTOR*, *NBPF10*, *NOTCH2*, *ZC3H11A*, and *KRTAP1-5*) [94]. Additional studies have identified the role of the MHC1 complex including *NLCR5*, *HLA-A/B/C* impacting NPC, and its critical role in EBV antigenic presentation [101]. Studying these specific germline mutations can help understand NPC pathogenesis and diagnose patients earlier.

### Somatic mutations

Because germline mutations are important for family considerations in both local and metastatic settings, we will cover them in this article. Somatic mutations and association with targeted therapies will be covered in the metastatic NPC article.

## Conclusion

Current research and the latest treatment advances in locally advanced NPC include induction vs adjuvant chemotherapy, which chemotherapeutic regimen, and the role of de-escalation. Research focusing on the role of EBV may guide aggressive treatment in those with elevated pretreatment and persistent EBV levels following treatment. With the important role of molecular biology, new and targeted therapies may offer personalized treatment. Similar to other cancers, somatic and germline mutations play an important role in NPC oncogenesis. The analysis of these mutations offers a new method of identifying familial cases earlier. Additionally, in the locally advanced setting studies, have been initiated evaluating VEGF, EGFR, and checkpoint inhibitors added to chemoradiation. We await the outcome.

**Fig. 1:**
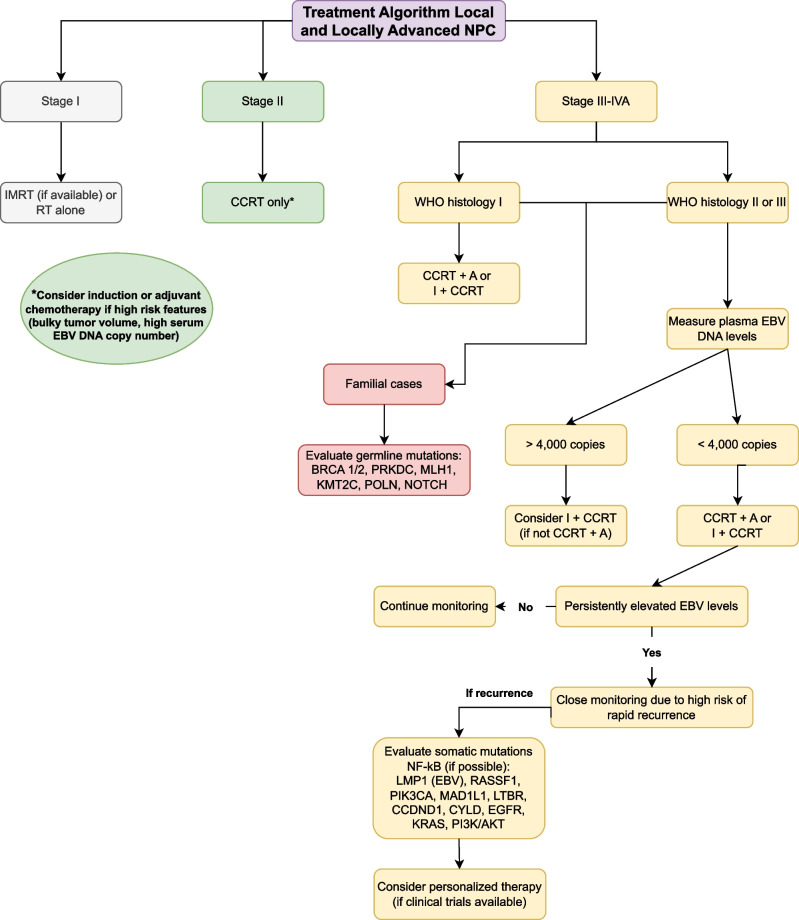
Treatment algorithm summary for local and locally advanced nasopharyngeal carcinoma.

This review has the goal of offering a comprehensive algorithm (Fig. 1) summarizing the management of patients with locally advanced NPC. Details regarding these treatments can be found in the specific sections of the manuscript.
